# Spaceflight Stressors and Skin Health

**DOI:** 10.3390/biomedicines10020364

**Published:** 2022-02-02

**Authors:** Wilhelmina E. Radstake, Bjorn Baselet, Sarah Baatout, Mieke Verslegers

**Affiliations:** 1 Radiobiology Unit, SCK CEN, Belgian Nuclear Research Centre, 2400 Mol, Belgium; eline.radstake@sckcen.be (W.E.R.); sarah.baatout@sckcen.be (S.B.); mieke.verslegers@sckcen.be (M.V.); 2 Department of Molecular Biotechnology, Faculty of Bioscience Engineering, Ghent University, 9000 Ghent, Belgium

**Keywords:** spaceflight, skin, microgravity, ionizing radiation, psychological stress, space simulation models

## Abstract

Traveling to space puts astronauts at risk of developing serious health problems. Of particular interest is the skin, which is vitally important in protecting the body from harmful environmental factors. Although data obtained from long-duration spaceflight studies are inconsistent, there have been indications of increased skin sensitivity and signs of dermal atrophy in astronauts. To better understand the effects of spaceflight stressors including microgravity, ionizing radiation and psychological stress on the skin, researchers have turned to in vitro and in vivo simulation models mimicking certain aspects of the spaceflight environment. In this review, we provide an overview of these simulation models and highlight studies that have improved our understanding on the effect of simulation spaceflight stressors on skin function. Data show that all aforementioned spaceflight stressors can affect skin health. Nevertheless, there remains a knowledge gap regarding how different spaceflight stressors in combination may interact and affect skin health. In future, efforts should be made to better simulate the spaceflight environment and reduce uncertainties related to long-duration spaceflight health effects.

## 1. Introduction

When the first humans ventured into space, it became clear that the space environment is unwelcoming for the human body. Weightlessness, or microgravity, is most obviously encountered in space. Additionally, not readily visible but vastly present, are higher levels of ionizing radiation. Finally, the high workload, social isolation, and confined area of the spacecraft adds another spaceflight stressor to cope with. In recent years, psychological stress has become a major concern for the overall health of the space crew. Together, these spaceflight stressors interact with human physiological systems and form a threat for the human body in the long run. Problems related to musculoskeletal deconditioning, microgravity-induced fluid shifts and cardiovascular issues are on the list of spaceflight-related disorders [1]. Still, plans for future long-duration interplanetary missions are in progress and in demand, which is why research into these spaceflight-induced defects and the development of possible countermeasures is warranted. 

**Figure 1:**
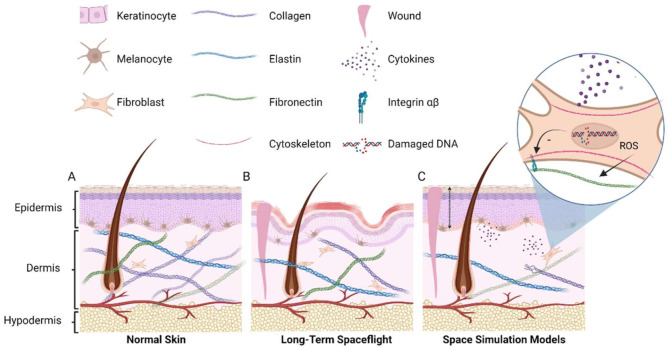
Schematic representation of healthy skin and spaceflight environment on the skin. (**A**) Simplified representation of normal skin tissue showing the epidermis consisting of several layers of keratinocytes, the dermis with extracellular matrix proteins secreted by fibroblasts, and the hypodermis. (**B**) Astronauts report sensitive skin depicted by a red epidermal layer, as well as small cutaneous wounds and delayed wound healing. Thinning of the epidermis has been found in some astronauts as well as loss of dermal matrix proteins with an increase in elastin/collagen ratio, loss of skin elasticity and reduced melanin content. Thinning of the dermis has been observed in mice. (**C**) In simulation models which mimic certain aspects of the spaceflight environment on Earth, loss of collagen proteins and delayed wound healing is shown after cortisol exposure. In addition, ionizing radiation affects dermal matrix gene expression, DNA integrity observed in fibroblasts, and leads to an increased expression of cytokines indicating an inflammation-like state of the skin. Epidermal thickness (dashed arrow) as well as cell number in the basal layer (presented in red) show linear energy transfer (LET)-dependent alterations. Furthermore, simulated microgravity has been shown to affect the gene expression of cytoskeletal components and delayed migration of fibroblasts pointing towards delayed wound healing. This figure was created using BioRender.

One of the organs that undergoes changes during spaceflight is the skin (see Figure 1). Accounting for up to 16 percent of the total bodyweight, the skin is the largest organ of the human body [2]. Its function is to protect the body from environmental hazards and, as such, acts as a protective barrier for the underlying tissues. To fulfill this function, the skin is composed of several layers, each with a unique role. The outer layer is called the epidermis and forms a tough and waterproof coat. Beneath the epidermis lies the second layer called the dermis. This softer layer consists of connective tissue and works as a cushion to protect the body from mechanical stress. Finally, the dermis is separated from underlying tissue by a layer of subcutaneous fat called the hypodermis. 

Forming the barrier between the environment and the internal body, the skin has to be able to adapt quickly and adequately to a wide variety of external stressors. Mechanical forces, as well as solar and thermal radiation, are all factors that can disrupt the proper barrier function of the skin. To be able to cope with different external stressors and maintain structure and functionality, the skin serves as a neuroendocrine system [3]. This complex interaction between the nervous-, endocrine- and immune system rapidly recognizes and integrates biological signals and coordinates skin stress responses with the aim of maintaining a proper barrier function vital for internal homeostasis [3,4,5].

Astronauts most frequently report skin-related problems, including itches, rashes, and dryness of the skin [6,7,8]. These issues are thought to be related to the environment of the International Space Station (ISS), in which the skin hygiene routine is limited to the use of wet tissue wipes and non-rinse shampoos and soaps. Other factors, such as temperature, air circulation and low humidity levels further contribute to increased incidences of skin infections, atopic dermatitis, and dryness and itching of the skin. Furthermore, shifts in skin microbiota have previously been indicated in a cohort of nine astronauts, showing a reduction in Proteobacteria associated with increased hypersensitivity and infections of the skin [9]. Finally, contact dermatitis is often reported, which is mostly due to irritating substances such as biosensors, tape or electrode patches. 

Additionally, small cutaneous injuries and a delayed healing of wounds is observed during spaceflight. Wound healing is a complex multistage and multicomponent process that starts with the inflammatory phase. Upon wound induction, a blood clot is formed to stop the bleeding, and inflammatory cells migrate to the wound site and start clearing the contaminating substances and microbes. During the next stage, the proliferating phase, fibroblasts, keratinocytes and endothelial cells migrate to the wound and start proliferating and remodeling the damaged tissue. The final stage, or remodeling phase, consists of wound contraction and tissue remodeling of the original structure and function, which can last for many weeks after injury [10].

The skin issues reported during spaceflight cause concern for space agencies and researchers. Therefore, attempts have been made to better comprehend the changes undergone by the skin and to find possible countermeasures to maintain healthy skin. One recent publication summarizes the spaceflight-induced skin alterations observed in astronauts [11]. However, in this review, we intend to give an overview of the research performed in space as well as on Earth, and summarize these findings that have helped to better understand the skin’s reaction to the space environment in response to the different stressors encountered in space. We discuss the status of skin research in space, the methodology used in studies to recreate the space environment on Earth, identify gaps in the literature and the weaknesses of such methodologies, and provide a future aim for spaceflight research.

## 2. Current Knowledge on Spaceflight-Induced Skin Health Effects

### 2.1. Human Spaceflight

Even though skin issues have often been reported by astronauts, to date, only a few studies have measured and documented skin alterations during long-term spaceflight. An overview of mission duration, sample sizes and the most important findings can be found in Table 1. A schematic representation of the observed effect of the spaceflight environment on the skin, either real or found in simulation models on Earth, is shown in Figure 1.

The importance of preserving healthy skin in space has recently been demonstrated in a case report showing skin sensitivity and erythema in gravity-dependent areas, such as pressure points on the back and the posterior part of the legs in supine positions, upon return after a one-year space mission [12]. The sensitivity was limiting the astronaut’s post-flight duties, and frequent breaks during reconditioning sessions had to be implemented due to pain. In fact, skin problems such as hypersensitivity were one of the most reported health issues on board the ISS (1.12/flight year), as well as injuries, frequent itching, and dryness complaints [6,7,8]. Other notable medical events were upper respiratory symptoms such as congestion, rhinitis and sneezing (0.97/flight year), followed by various other symptoms such as cold sores, ear problems, and infectious symptoms (>0.3/flight year). This increase in skin sensitivity, reported as rashes during flight, might be a consequence of a dysregulated immune system in microgravity [13,14,15]. Furthermore, increased skin sensitivity, skin thinning and delayed wound healing, as well as increased susceptibility for skin infections, have been reported [16].

A study carried out on one astronaut indicated a loss of elasticity of the skin post-flight. These measurements thereby confirmed subjective reports on skin issues by showing degradation of the dermal matrix, thinning of the epidermis, and coarsening of the skin surface, which could indicate a decreased turnover time of epidermal cells. Epidermal thinning was later confirmed in two other astronauts who participated in long-term spaceflight, showing more than 10% thinning of the epidermis post-flight compared to pre-flight and a decreased melanin content. Furthermore, significant changes in the dermal extracellular matrix were found with an increase in collagen levels post-flight [17].

Interestingly, recent investigations into skin changes in a larger cohort of six astronauts showed contradicting results. Post-flight mean skin elasticity, skin density and thickness were unchanged compared to pre-flight. Measures of skin microcirculation showed a post-flight decrease in oxygen saturation and relative capillary hemoglobin concentration, although these measures stabilized over time. Moreover, an improvement of the skin barrier function, measured as transepidermal water loss (TEWL) and hydration, were observed. These data were supported by subjective reports of astronauts indicating better skin health during their missions [18]. One possible explanation for this apparent contradiction between the recent study [18] and previous studies could be the improvement of in-flight conditions, with adjusted nutrition and exercise devices positively influencing skin health. Alternatively, pre-flight activities might overstress the skin and worsen skin health where, for instance, pre-flight travel to different countries significantly affects hydration levels of the epidermis. Stabilization of TEWL and hydration measures in space indicate the benefit of standardized conditions in space, which might improve already damaged skin [16,18,19]. Nevertheless, variation due to the small sample size (*n* = 6) and differences in time points for data collection were observed between astronauts. Even though mean skin health values might have improved for TEWL and hydration, individual values varied between astronauts with lower in- and post-flight hydration values in some individuals [18]. Further investigation, including larger sample sizes, is thus needed to better understand the current results and individual differences in skin health.

### 2.2. Animal Studies and In Vitro Data

From previous examples of skin reactions in space, it is clear that more research is needed to determine the effects of long-term space missions on the skin. Currently, missions last up to approximately six months, with few cases of longer missions. However, compared to a three-year space mission to Mars, ISS-space missions are too brief to achieve a full understanding of skin adaptation in space. To overcome this problem, scientists have turned to animal and cellular models.

Thanks to astromice, who resided in the ISS for three months—the equivalent of approximately 7 years in human life—we now know that dermal thickness reduces as much as 15% during this period in space. A probable explanation for this reduced thickness is an altered collagen turnover, with higher degradation of newly formed pro-collagen [20]. However, astromice flown to space for only 13 days already show alterations in their gene expressions profiles related to oxidative stress and extracellular matrix (ECM) remodeling. Interestingly, in these space-flown mice, corticosterone levels were shown to be increased compared with the ground controls. Consequently, the authors argue that the effects of corticosterone on physiological processes could underlie the observed shifts in biological and metabolic homeostasis [21].

An increased inflammation response [22] and altered collagen synthesis [23] have also been observed in fibroblasts flown to space. In order to measure DNA damage and inflammation response in fibroblasts aboard an unmanned Foton-M3 capsule, specialized hardware was developed which enabled the researchers to successfully cultivate human-derived dermal fibroblasts, the major cell type of the dermis, in space. During their 5-day space mission, fibroblasts increased the expression of inflammatory cytokines compared with the ground controls cultured in identical hardware, suggesting an inflammation-like process is likely to be induced in these skin-derived fibroblasts shortly after being exposed to the space environment [22]. Finally, space-flown fibroblasts showed increased cell density in confluent cultures that had been in space for 14 days, compared with the ground controls. Gene and microRNA expression profiles indicated increased cell growth as a result of the activation of the nuclear factor kappa-light-chain-enhancer of activated B cells (NF-κB) pathways, as well as other growth factors.

**Table 1:** Overview of studies measuring skin alterations during long-term spaceflight.

	N *	Mission Duration	Measures	Findings	Reference
Human data	1	340 days	Clinical case report	Post-flight erythema and skin sensitivity in gravity-dependent areas;	[12]
				Persistence of skin problems for 6 days;	
				Successful treatment with nonsteroidal anti-inflammatory drugs, gabapentin, hydrotherapy, and massage.	
	1	6 months	Hydration stratum corneum (Corneometer^®^), TEWL (Tewameter^®^), skin surface structure (SkinVisiometer^®^), skin elasticity (Cutometer^®^), skin ultrastructure (20-MHz ultrasound, DermaScan^®^)	Delayed epidermal proliferation	[16]
				coarser skin surface post-flight;	
				Significant loss of elasticity of the cutis post-flight;	
				Degradation of fiber structure in the cutis post-flight.	
	2	long-term	Hydration stratum corneum (Corneometer ^®^), TEWL (Tewameter^®^), skin topography (VisioScan^®^), skin elasticity (Cutometer^®^), skin density (20 MHz ultrasound), skin morphology and metabolism (Multiphoton tomography MPTflex™).	Thinning of the epidermis post-flight	[17]
				decreased melanin concentration post-flight;	
				Increased elastin/collagen ratio post-flight.	

	6	>4 months	Hydration stratum corneum (Corneometer^®^),	Improvement in skin hydration and barrier function;	[18]
			TEWL (Tewameter^®^),	No apparent changes in skin surface;	
			skin surface profile (VisioScan^®^),	Unchanged skin density, thickness and elasticity post-flight.	
			skin elasticity (Cutometer^®^),		
			skin density and thickness (20-MHz ultrasound, DermaScan^®^),		
			capillary blood flow, flow velocity, hemoglobin concentration, hemoglobin oxygenation (O2C, LEA Medizintechnik GmbH).		
Rodent data	3	3 months	Stained tissue sections for determining thickness of the dermis, hypodermis and panniculus carnosus, and number of growing hair follicles, hydroxyproline assay to determine skin collagen content and extractability, microarrays and RT-PCR.	Significant reduction in dermal thickness post-flight;	[20]
				Increased collagen turnover;	
				Significant modulated ECM gene expression;	
				Increased number of hair follicles in anagen phase accompanied by a high expression of specific hair follicles genes	
				Modulation of transcripts encoding proteins related to striated muscle homeostasis.	
	6	13 days	RT-PCR and microarrays, high-throughput metabolomics profiling, immunofluorescence staining for oxidative damage marker on skin tissue sections.	Alteration of expression of genes related to production and metabolism of ROS; Alteration of expression of genes related to production and metabolism of ROS;	[21]
				Altered expression of genes encoding MMPs involved in ECM remodeling.	

* The number of test subjects or animals included in the study. ECM, extracellular matrix; MMPs, matrix metalloproteinases; ROS, reactive oxygen species; RT-PCR, reverse transcriptase-polymerase chain reaction; TEWL, transepidermal water loss (TEWL).

### 2.3. Limitations of Spaceflight Studies

As accessing space is currently still costly, and obtaining data from astronauts is complicated and subject to several logistic restrictions, scientists have few opportunities for conducting their experiments in orbit. Unmanned capsules are a useful way of accessing space, though the development of hardware, such as life-support systems for animal studies, is time consuming and prone to failure. Because of these restrictions, sample sizes of astronauts, as well as space-flown in vivo and in vitro experiments, are usually small. For example, the largest cohort of astronauts who participated in skin research consisted of only six subjects [18]. Furthermore, of the six astromice who were flown to the ISS, scientists were only able to collect data from three, as the other mice died before the end of the mission [20]. Because of the struggles related to spaceflight research, methods have been developed to simulate the space environment on Earth. As such, in vivo and in vitro study designs are being used, each focusing on a specific spaceflight stressor of which the physiological effect can be studied in detail. In the next section of this review paper, we describe some of the ground-based methods that have been used in order to investigate specific aspects of the spaceflight environment on skin physiology.

## 3. Skin and Wound Healing Models

Arranged with an elongated fusiform morphology, fibroblasts are the most abundant cell type found in connective tissue. Their wide array of functions in skin-ECM secretion, cytokine expression, epithelial differentiation and wound healing make them important for normal skin functioning, and their involvement in several diseases has been indicated [2]. Skin biopsies from patients provide an easy, though invasive, method to obtain primary fibroblast cultures, which are easily maintained in vitro [24]. As skin fibroblasts are crucial for skin homeostasis, a lot of diversity is shown within this cell type, and depending on biopsy site and differentiation state, differences in morphology and functionality are shown [25,26]. Because of their diversity, mono-cultures of fibroblasts can be used to study several aspects of wound healing including migration, proliferation, and protein synthesis [27].

In the skin, fibroblasts provide a support for keratinocytes in the epidermis. The close anatomical proximity between fibroblasts and keratinocytes is crucial for proper skin homeostasis, and provides the possibility of communication between both cell types either by direct cell–cell interactions, or dual paracrine signaling. This interaction is essential in several skin processes including regulating the different stages of wound healing [28,29], and co-cultures can be used to study complex cell–cell interactions during the wound-healing process [27]. Monocultures of keratinocytes or fibroblasts fail to represent the complex interaction and spatial organization between both cell types, hindering a translation of in vitro data to the human skin. 

To overcome this issue, in vitro skin tissue equivalent models have been developed [30]. In these skin models, fibroblasts are seeded into ECM components such as collagen, after which keratinocytes are seeded on top, a few days later. Finally, keratinocyte differentiation is induced by exposing the culture to air, and a cornified epidermis is formed. These organotypic skin models have advanced dermatological research by improving our understanding of the complex interaction between keratinocytes and fibroblasts. In addition, more complex models of skin equivalents have been developed by merging together a broad range of relevant cell types, such as melanocytes, hair follicle cells, Langerhans cells, and endothelial cells, as well as peripheral neurons (for a full overview, readers are referred to [26]). Finally, recent skin-on-a-chip models are being developed, which incorporate in vitro 3D skin models inside microfluidic devices allowing for perfusion mimicking vascularization. This technology allows researchers to control factors such as fluid flow, mechanical forces, and chemical application, and is of special interest for studying drug development and cosmetic testing [31], but could also hold interesting opportunities for space-related studies [32].

Besides in vitro models, several animal models can be used to study skin health effects. Pigs’ skin show morphological similarities to human skin; for this reason they have been used to study radiation effects on the skin [33]. The skin’s function related to wound healing can be studied in a variety of animal models including rat, mouse, rabbit and guinea pig models. Depending on the specific research interest and phase of wound healing targeted (e.g., epithelization, angiogenesis, dermal reconstruction, wound contraction, granulation), different animal models and wounding methods can be used. An extensive overview of such models falls outside the scope of this review, but the reader is referred to [27] for a comprehensive overview.

## 4. Ground-Based Space Simulation Models and Their Effect on Skin Health

### 4.1. Simulated Microgravity

When an object is in orbit, the centrifugal force generated by the launch of the spacecraft counteracts the gravitational force towards the Earth. As a result, a state of constant free fall around the Earth is maintained. On board, the crew experiences this constant free fall as weightlessness. Although this weightlessness provides a unique environment for multiple fields of research, it limits the functioning of the human body, which has evolved in Earth’s 1 g environment. Adaptation to this microgravity environment is possible for some physiological systems. For example, most astronauts experience symptoms of space-motion sickness during the first days in space. These symptoms are believed to result from conflicts in vestibular and visual information, and after an adaptation period symptoms ease and astronauts seem to be fully adapted to the new microgravity environment [34]. This adaptation is, however, not evident for most other physiological systems which, without the use of proper countermeasures, show a gradual deterioration during long-term space travel [1].

Brief periods of microgravity can be obtained on Earth using drop towers or parabolic flight [35]. However, these brief periods are inadequate to observe skin physiological changes. To enable researchers to investigate these effects, methods are used that mimic the effects of a lacking gravity vector on the human body. For instance, the unloading of the body and the induction of the head-ward fluid shift, as observed in space, can be recreated in head-down bed rest (HDBR) [36] studies in humans, or hindlimb unloading models used for rodents [37]. Additionally, to simulate the microgravity for in vitro studies, the Random Positioning Machine (RPM) can be used. This machine rotates with a random velocity and direction around three axes, thereby averaging the total gravity vector to zero [38].

When exposed to this simulated microgravity environment, skin cells adapt their form and function [39,40,41]. This is thought to happen through the mechanosensitive cytoskeletal structures which, when confronted with alterations in mechanical stress, initiate a transduction process which results in a biochemical response [42,43]. This has been exemplified in fibroblasts and keratinocytes that show changes in gene expression after exposure to simulated microgravity. More specifically, reduced gene levels of β1-integrin, fibronectin and actin filament have been observed, as well as the downregulation of gene coding for stress response, cytoskeleton organization, and adherent junctions [1,40,44,45].

ECM remodeling, a feature of fibroblasts which is crucial for wound healing, has been shown to be affected during exposure to simulated microgravity. The gene expression of different collagen types, fibronectin, laminin, and vitronectin, as well as matrix metalloproteinases (MMPs) was diminished in cells exposed to simulated microgravity [46,47]. However, in another study, protein levels of fibronectin were found to be increased in simulated microgravity-exposed fibroblast cultures [47]. Of note, the upregulation of expression levels of the α1 chain of collagen type I (COL1A1) has been indicated in co-cultured fibroblasts and keratinocyte spheroids [48]. This highlights the complex relationship between microgravity and ECM remodeling in skin cells. Indications of cytoskeletal remodeling have also been found after simulated microgravity exposure. A significant increase in integrin receptors (α5β1), as well as alpha-actin, have been detected in fibroblasts [47]. Furthermore, reduced expression levels of alpha smooth muscle actin (α-SMA) and epithelial cadherin, proteins involved in cytoskeletal reorganization and the formation of adherent junctions, have also been shown in fibroblasts exposed to microgravity [39]. As these proteins are essential for cellular adhesion and migration, it is no surprise that the study found these functions to be impaired in fibroblasts exposed to modeled microgravity, although increased cell adhesion after simulated microgravity exposure in fibroblasts has been indicated as well [47]. On the contrary, in keratinocytes, migration behavior was enhanced during microgravity exposure [41] which could suggest a cell-type-dependent altered migratory behavior in microgravity. Finally, the reduced expression of α-SMA has further been indicated in fibroblasts grown on 3D collagen matrices in a simulated microgravity environment [46]. These findings point to an impaired fibroblast differentiation possibly explained by the reduced translocation of Smad2/3 into the nucleus.

As can be concluded from this, the comparison of studies investigating the effects of simulated microgravity on wound healing features, such as ECM, cytoskeletal remodeling and cell migration, is not straightforward. Besides the type of cell culture used, reasons for cell-type-specific differences in migration behavior after the microgravity exposure of different cell types could be found in the applied methodologies. For instance, while using a scratch assay to study spontaneous migration upon injury, fibroblasts were only scratched 72 h after microgravity exposure and measured for the next 48 h. In contrast, keratinocytes were exposed to modeled microgravity after wound induction, measuring the migration capacity during simulated microgravity exposure [39,41]. While the latter methodology is much more challenging, it more closely represents the migration capability occurring in space. Other differences between the mentioned studies, such as the type of hardware used, RPM vs. a Rotary Cell Culture System (RCCS), duration of exposure, and medium composition might have contributed to observed differences as well [39,41,46,47,48]. To make better predictions on the wound healing capabilities of skin cells in space, a uniform protocol that is as close to the real space environment as possible needs to be developed.

### 4.2. Ionizing Radiation

Without the protective shielding of Earth’s atmosphere, astronauts are exposed to higher levels of ionizing radiation, posing another health risk related to the spaceflight environment. Galactic cosmic ray (GCR) particles derived from supernova explosions far outside of our solar system provide a continuous background of high- charge and energy nuclei (HZE). These HZE particles are highly ionizing along their tract, and can penetrate deeply into matter. Additionally, the incidence of solar particle events (SPE) creating a high flux of protons and alpha particles, as well as trapped protons and electrons in the Van Allen belts complete the radiation environment to which astronauts are exposed [49]. Although these particles create an increased risk of exposure to high doses, especially for the skin, relatively easy shielding can prevent any major damage. Health effects from exposure to the continuous background of GCR are mostly stochastic. However, data of human exposure to this type of radiation is lacking; therefore, risk-assessment is unreliable and the uncertainty of disease development such as cancers, cataracts or damage to the central nervous system is large. For this reason, space radiation is considered the main obstacle for deep space exploration [50,51,52].

To mimic space radiation on earth, researchers make use of high-energy ion beams at accelerator facilities, of which only a handful are available today. Single ion beam exposures to animal models and cells have greatly contributed to our understanding of space radiation health effects. Nevertheless, uncertainties about risks for mortality or developing fatal cancers due to exposure to space radiation on long-term deep-space missions remain high [53]. As a result, risk estimates considered for lunar and Mars space missions exceed space agencies’ limits of risk for exposure-induced death for radiation carcinogenesis [52]. To better understand the associated risks and reduce uncertainties, ground-based experiments using heavy ion accelerators to mimic space radiation can provide precious insight into the biological effects of exposure, as well as answer questions related to health effects, shielding, and countermeasure development [54].

Although the use of these mono-energetic single-ion beams can provide crucial insight into the effects of exposure to HZE particles, and thereby improve risk assessment, the actual space radiation environment is composed of a wide variety of ions at different energies [52]. As a result, single-ion exposure is limited in approximating the radiation environment of deep space, and underestimates the possible synergistic effects of different particles and energies. Researchers at the NASA Space Radiation Laboratory (NSRL) have developed a GCR Simulator which overcomes this limitation by using fast beam switching to be able to expose samples to a variety of ion beams and, as such, gives a better representation of the space radiation spectrum to be experienced by astronauts in deep-space (for more information, readers are referred to [55]). Efforts for recreating the space radiation environment on European ground have resulted in the development of a hybrid active-passive simulation approach designed for the GSI/FAIR facility. Here, a mixed field is created by actively varying the energy of heavy ion beams, combined with the use of beam modulators [56]. Future experiments carried out at these facilities will provide a better understanding about space radiation-induced health effects, and as such reduce the risk uncertainties linked to deep-space missions.

Due to their high ionizing power, high charge and energy (HZE) particles contribute significantly to the radiation risks in space [52,57]. When these HZE particles interact with cells, an increased damaging power of these particles is observed compared with less densely ionizing radiation. Of note, besides the contribution of linear energy transfer (LET) to increased cell killing, the ion species itself can have a significant effect as well, as shown by Tsuruoka and colleagues (2005) who irradiated fibroblasts with heavy ion beams of carbon, neon, silicon and iron ions at different energies (see Table 2, for an overview of exposure conditions), and indicated different relative biological effectiveness (RBE)-LET curves for cell killing depending on ion species. These observations suggest that differences in energy deposition along the tracks of different ions have a distinct biological effect on cell killing [58]. 

For deep-space exploration the high energy of the GCR particles, with a peak in the energy spectrum around 1 GeV/u, causes difficulties for shielding and increases the risk of astronauts developing cancers in the long term [52]. This is believed to result from the high potency of high-LET radiation to create complex DNA damage when traversing a cell nucleus. Faulty or unrepaired DNA breaks lead to chromosomal aberrations and an associated increased risk of developing cancers [59,60]. Even at fluences of less than one HZE particle per cell (see Table 2 for an overview of exposure conditions), increases in chromosomal aberrations have been found in fibroblasts, supporting the possibility of a non-targeted bystander effect, and having important implications for radiation protection for astronauts [61]. Nonetheless, despite the sensitivity of the skin to ionizing radiation, and the fact that skin cancer was the first cancer which was associated with exposure to ionizing radiation, radiation-induced skin cancers are often not considered as high risk, and are easily treatable with current medical care available on Earth (for a detailed description readers are referred [62]).

Besides inducing damage to the DNA, HZE particles have also been shown to influence gene expression. Gene expression changes were found in fibroblasts irradiated with high-LET iron ions (see Table 2 for an overview of exposure conditions), showing a stronger effect in fibroblasts irradiated with higher LET as compared to lower LET silicon and neon ions. These results suggest an LET-dependent effect on the magnitude of induced expression changes. However, differentially expressed genes were comparable to those observed after X-irradiation, and were shown to be mostly involved in cell-cycle arrest and apoptosis [63]. Furthermore, in a rat keratinocyte model, the upregulation of 69 genes was found after irradiation with 3 Gray (Gy) iron ions (1 GeV/u) (see Table 2 for an overview of exposure conditions) compared with non-irradiated keratinocytes, particularly involved in cell cycle arrest, mitosis and DNA repair [64]. Dose-rate effects (see Table 2 for an overview of exposure conditions) have also been shown to alter gene expression in irradiated mouse skins. For example, with a total dose of 0.25 Gy, a low dose-rate (25 cGy/hour) induced greater changes in gene expression related to oxidative stress as compared to a high-dose-rate (25 cGy/min) regimen. Of interest, in the low-dose-rate group, ionizing radiation induced the up- and downregulation of genes involved in ECM structural components. The authors propose that the altered gene expression profiles related to ECM remodeling increased the expression of antioxidants and reactive oxygen species (ROS) production, leading to a shift in skin homeostasis [65].

To understand the effects of radiation on skin health, 3D human skin equivalent (HSE) models were irradiated (see Table 2 for an overview of exposure conditions) with both X-rays and high-LET carbon ions (100 keV/µm). Both dose and radiation type showed an effect on the expression of inflammation markers, showing the strongest upregulation of cytokine release after high doses (2 and 10 Gy) of X-rays. Contrarily, irradiation with carbon ions at 2 and 0.5 Gy induced only minor changes in cytokine expression [66]. Different particles (oxygen, silicon and iron) and fluences (at 3.6 × 10^−4^ ions per µm^2^ for low vs. 1.1 × 10^−3^ ions per µm^2^ high) (see Table 2 for an overview of exposure conditions) have been used to establish the effects related to radiation quality on human skin tissues. The skin model showed the influence of charged particles on the proliferation of cells in the epidermal layer of an HSE model, although no clear pattern based on radiation quality was established. Fluence dependence on proliferation rate could be established for both oxygen and iron ions. A decrease in proliferation was shown at higher fluences. In contrast, an increase in proliferation rates could be observed for lower fluences. Similar to this, the proliferation of cells in the basal layer of the epidermis was enhanced after a lower dose (0.5 Gy) of X-irradiation, while no effects were found in the proliferation rate after exposure to carbon ions at a lower dose (0.5 Gy). However, exposure to carbon ions at 2 Gy showed reduced proliferation rates of epidermal keratinocytes [66]. Interestingly, temporal effects differed for all ion species, making it hard to establish any patterns. Furthermore, proliferative changes in the dermal layer were minor, showing only decreases in proliferation upon exposure to iron and silicon ions 24 and 48 h after irradiation [67].

Finally, thickness of epidermal layers has been suggested to be influenced by ionizing radiation, showing LET-dependent behavior. For instance, increased epidermal thickness after exposure of HSE to oxygen ions at doses of 0.1–3 cGy (see Table 2 for an overview of exposure conditions) has been indicated. However, when the HSE was irradiated with higher LET particles, this response was altered showing thinning of the epidermal layer after exposure to silicon ions at 60 keV/µm, and only minor changes in epidermal thickness for iron ions at 174 keV/µm [67]. Cell numbers in the basal layer have been shown to both increase and decrease over time as a function of LET. Exposure to lower LET ions (oxygen ions, 18 keV/µm) shows an increase in basal cell numbers while the opposite has been found after exposure to higher LET ions (silicon and iron ions, 60 and 174 keV/µm) [67].

Taken together, besides the destructive effects of ionizing radiation on DNA and the increased risk for the development of cancers later in life, exposure to ionizing radiation, even at lower doses, can harm a healthy function of the skin. Hereto, radiation quality, ion species, LET, total dose and dose-rate have all shown to induce DNA damage, to affect cell survival, gene expression, cell proliferation and differentiation, and induce inflammation. Finally, as the skin is a dynamic organ with high adaptability, temporal effects are usually an important factor to consider when observing skin changes. Consequently, the interpretation of the effects of ionizing radiation on skin health is not straightforward, and strongly depends on the study design. Hence, to achieve more reproducible and comprehensive data, the above-mentioned factors should be taken into account and precisely reported, which is, in fact, a general concern in radiobiological studies suffering from poor replicability [68]. Furthermore, to find meaningful answers as to how ionizing radiation in space can influence human health—and to develop effective countermeasures—efforts should be taken to mimic those factors shown to influence the biological endpoints observed in space as precisely as possible.

### 4.3. Psychological Stress

Besides continuous exposure to microgravity and higher levels of ionizing radiation, astronauts experience increased levels of psychological stress. Social isolation, living in a confined environment, and a high workload can all contribute to elevated cortisol levels. The hypothalamic pituitary adrenocortical (HPA) axis and its function to release cortisol are crucial for individual survival. Glucocorticoids mobilize energy resources through catabolic mechanisms which are needed for responses to either internal or external stressors, thereby promoting homeostatic adaptation [69,70]. When presented with a stressful event, specific neurons in the brain’s limbic system activate and secrete releasing hormones. Increased levels of corticotrophin-releasing hormone (CRH) and arginine vasopressin (AVP), released by these neurons, in turn stimulate the secretion of the adrenocorticotropic hormone (ACTH) by the hypothalamus. This release of ACTH then promotes the adrenal cortex to produce and secrete glucocorticoid hormones [69].

In healthy individuals the HPA axis activity and release of glucocorticoids are precisely controlled by negative feedback loops and follow a circadian rhythm [71]. Acute stress response is characterized by a temporary increase in ACTH and cortisol levels that return to baseline once the stressful event is eliminated. However, in the case of sustained stress, the negative feedback control becomes dysregulated and chronic upregulation of HPA axis activity induces high levels of circulating glucocorticoids [69]. This chronic stress is considered an important risk factor for the development of diseases related to autonomic, cardiovascular, gastrointestinal and immune system dysfunction [72]. Increased levels of cortisol have been measured in astronauts after both short- and long-term durations of spaceflight [73,74,75,76,77], indicating the need to further unravel health risks, including those to the skin, associated with increased cortisol levels in space.

Simulation models that are used to investigate the effect of chronic stress on the skin are based on the elevation of glucocorticoid levels. In rodents, chronic stress and rise in corticosterone can be induced by the chronic administration of exogenous glucocorticoids; this can be achieved by mixing it with drinking water, injecting slow corticosterone releasing pellets into the animals [78], or by presenting a stressor to the animal which will result in an HPA-axis response and release of glucocorticoids. Such models are based on physical stressors such as chronic restraint or immobilization, maternal separation, cold shock, food deprivation, or by inducing variation in the day–night cycle [79]. To investigate the effects of glucocorticoids on cellular function in vitro, simulation of chronic stress is often achieved by administrating soluble glucocorticoids to the cell culture medium.

In the skin, local stress responses are regulated by the skin equivalent HPA-axis. Dermal fibroblasts that are exposed to CRH increase proopiomelanocortin (POMC) gene expression and ACTH production which stimulate the production of corticosterone [5]. In a similar fashion, human body hair follicles rapidly produce cortisol upon exposure to pain or cold [80]. Dysregulation of this skin equivalent HPA axis has been suggested to lead to a series of inflammatory or autoimmune skin disorders [81]. The cortisol-activating enzyme 11ß-hydroxysteroid dehydrogenase (11ß-HSD) catalyzes the interconversion between inactive cortisone and active cortisol in cells. Reduced levels of 11ß-HSD-1 have been found in skin tumors and inflammatory skin diseases. Upregulation of cortisol by 11ß-HSD-1 decreases cell proliferation in keratinocytes and fibroblasts and delays wound healing. Increased levels of this enzyme are found in the elderly, suggesting an important role of 11ß-HSD-1 in age-related skin atrophy [82,83].

Furthermore, the application of glucocorticoids on the skin are known to effectively treat inflammatory skin disease; however, this can induce skin atrophy in cases of long-term usage [84]. Applying glucocorticoid crèmes twice a day for 3 to 4 weeks has shown to lead to thinning of the epidermal and dermal layer, decreased microvasculature and keratinocyte size, and the loss of barrier function [85]. Skin atrophy is also one of the manifestations found in patients suffering from Cushing’s syndrome that results from prolonged exposure to excess endogenous glucocorticoids. Other skin manifestations are bruising, purple striations, and delayed wound healing [86]. The reduced proliferation of keratinocytes and fibroblasts, as well as the disrupted synthesis of skin lipids, contribute to this glucocorticoid-induced skin atrophy. Besides, the function of fibroblasts to produce ECM proteins is severely hampered by glucocorticoid application [84,87]. Collagens are the main component of the ECM, and dermal fibroblasts play a major role in the production and degradation of collagen fibers, thereby providing healthy skin with tensile strength [2]. The drastically reduced expression of α1(I) and α1(III) collagen genes, as well as the low synthesis of tropocollagens type I and III, have been indicated in mouse skin treated with glucocorticoids, signifying the sensitivity of these genes and proteins to glucocorticoid treatment [88].

In addition, psychological stress can impact the healing of the skin, resulting in a delayed wound-healing process [89]. Chronic psychological stress, as result of caregiving for the elderly, during exam periods, marital conflicts, or for in mice, during restraint, has been linked to slower wound healing. This is possibly due to the suppressive effect of glucocorticoids on inflammatory cytokine expression during the initial inflammatory phase of wound healing (data reviewed in [90]). The reduced proliferation of keratinocytes and fibroblasts as result of increased 11ß-HSD-1 expression and thus local cortisol can further contribute to delaying the wound-healing process during the proliferation phase [91]. The inhibition of this enzyme has been shown to speed up wound healing in normal and aged mice, and prevent collagen degradation as a result of an age-related increase in local cortisol expression. Inhibiting this enzyme thus shows a potential target for promoting tissue repair in the elderly [83].

Taken together, data from animal and cellular studies as well as human disease models show a clear impact of cortisol expression on skin function. Nevertheless, only recently researchers have started to investigate the mechanisms involved in glucocorticoid-induced skin atrophy, which will lead to better insights into the negative impact of cortisol on skin cells.

### 4.4. Combined Exposure Effects on Skin Models

As has been indicated by Hada and colleagues (2019), the simultaneous exposure of fibroblasts to simulated microgravity and X-rays or carbon ions increased chromosomal aberrations. Using a system where the synchronization between RPM and heavy ion beam allows samples to be irradiated while they are in simulated microgravity has revealed that both simple and complex chromosome exchanges were increased in samples exposed to combined simulated microgravity and carbon ions, compared with static irradiated samples, suggesting the interactive effect of microgravity and cosmic radiation on chromosome aberrations [92]. Interaction effects, although not synergistic, of simulated microgravity and ionizing radiation, have also been indicated in murine fibroblasts continuously exposed to simulated microgravity and a mixed radiation field of neutrons and gamma-rays. Gene expression was shown to be altered after simulated microgravity or ionizing radiation treatment, associated with cytoskeleton remodeling and the oxidative stress response. However, gene sets in the combined exposure group did not include those found in the single exposure treatment groups, suggesting a complex interaction of both spaceflight stressors on gene expression [44].

Observing altered skin cell responses after exposure to combined simulated spaceflight stressors, compared with single exposure, clearly shows the challenge researchers face in unravelling the effects of spaceflight conditions on the skin. Hereto, the lack of studies combining multiple spaceflight stressors, in particular the inclusion of psychological stress, shows the need for combined exposure models in future research designs. It is only then that the spaceflight environment will be most optimally mimicked, which would drastically increase our knowledge of underlying biological pathways, and thereby improve the risk assessments associated with future interplanetary space missions.

## 5. Future Perspectives and Conclusions

From all the above, we can clearly conclude that simulated microgravity, ionizing radiation, and stress hormones, can influence the proper functioning of the skin, and as such compromise the skin’s vital functions. Data obtained from astronauts related to wound healing, skin thickness and barrier function are often contradictory, which might be due to inter-individual differences in skin sensitivity and insufficiently large sample sizes. Besides data from astronauts, mouse studies and cell culture experiments have also been conducted in space, and support the findings of dermal atrophy and increased inflammation after exposure to the spaceflight environment.

Of note, skin measurements in astronauts were carried out on different locations on their inner forearms. Skin thickness, barrier function, and hydration have been shown to differ depending on body location, as well as age and gender [93,94,95]. Future studies could include multiple body locations to obtain a better understanding of location-specific changes in skin during spaceflight.

With intentions of embarking on deep-space missions, how prolonged exposure to the spaceflight environment will influence astronauts’ health is of increasing concern. ISS studies are mostly lasting up to six months (with a few exceptions). In some astronauts, this relatively short stay in space already affects skin health. It is currently unknown how these skin alterations will evolve during deep-space missions to the moon or to Mars. Besides, with the growing interest of commercial and touristic spaceflight, proper health monitoring in space is becoming increasingly important. As such, skincare related to cleaning and moisturizing, as well as reducing injuries obtained from hardware such as spacesuits, should be optimized. Furthermore, a dysregulation of the immune system in microgravity can further contribute to the symptoms of skin sensitivity and rashes, and can delay wound healing [13,14,15,96]. Therefore, better understanding of immune function during long-term spaceflight, and how this may contribute to observed skin sensitivity, is desirable. Finally, with regard to wound healing, it is still poorly understood how this complex multi-phase process is affected by the spaceflight environment. As can be concluded from a recent review, it is expected that different wound healing phases are differently affected by the space environment, with possible impaired wound healing as an outcome. To better understand wound healing in the spaceflight environment, future spaceflight studies should investigate this important skin function in more detail [96].

In addition to spaceflight studies, there is a growing need for a more mechanistic understanding of the individual effect of space stressors on cell functioning, for which ground-based animal and cell-based studies are particularly interesting. However, little research has been performed so far to understand how spaceflight stressors interact and influence proper skin functioning, as well as to understand how we might overcome or prevent certain stressor-induced skin defects. In light of this, besides using simulation studies of individual spaceflight stressors, efforts should be made to combine such simulation models, as effects of spaceflight stressors on overall cell function are believed to be complex, and not simply additive. A deeper understanding of how spaceflight stressors affect cell functioning does, however, require the development of standardized tests. Currently, a lack of reporting specific study aspects, such as RPM settings and sealing of cell culture vessels in simulated microgravity experiments, and irradiation characteristics in radiobiology experiments, makes the comparison of studies problematic. Differences in cell culture techniques (2D, 3D, co-cultures) further contribute to the heterogeneity of spaceflight data. In the future, efforts should be made towards the standardization of spaceflight studies (either real or simulated), which could, for instance, be based on an international consortium.

With regard to the investigation of isolated spaceflight stressors, effects of increased psychological stress levels and effects of elevated cortisol levels on the skin and other physiological systems have so far been under-investigated in spaceflight-related studies. This is concerning, as psychological stress levels are expected to increase during future interplanetary space missions, as a result of a longer duration of isolation and confinement, which will put more emphasis on crew social dynamics. Moreover, the growing distance from Earth will bring an increased psychological burden, as crews will be entirely self-reliant. Furthermore, communication with Earth will become significantly delayed during such missions, which might induce feelings of isolation and separation from loved ones. Besides, the increase in space tourism will expose more individuals to stressful space environments in the future. One can speculate that stress response in these untrained space tourists might be higher, and as such put these tourists at higher risk for developing stress-related symptoms, compared with astronauts who received training for multiple years.

The relative ease of obtaining and maintaining fibroblast cultures, together with the fact that they play a crucial role in various skin processes, makes this a suitable method to simulate the combined spaceflight environment. Indeed, this skin cell type is ideal to study the effect of the spaceflight environment, for instance cytokine and growth factor expression, ECM synthesis and remodeling, and in vitro wound healing. Yet, as previously discussed, the complexity of the skin and the interaction between fibroblasts and other skin cells hinders a translation of monoculture experiments to the in vivo situation. Complex models of organotypic skin cultures and skin-on-a-chip methods would therefore be more desirable, although we are still faced with experimental challenges and technical limitations with regard to exposing these set-ups to the simulated spaceflight environment.

To better understand the relation between spaceflight stressors on skin physiology and to develop effective countermeasures, in vitro studies should eventually be validated in vivo, for which animal models can be used. Hindlimb unloading in rodents is a commonly used model to mimic the microgravity-induced head-ward fluid shift, and has shown to induce bone loss also observed in space, which was intensified when combined with low-dose X-ray exposure [37]. Combining this model with cortisol exposure, as well as chronic low dose, and low-dose-rate exposure to ionizing radiation, as described by Acharya and colleagues [97], will complete the simulated spaceflight environment and could lead to significant new insights.

To date, treatment for observed skin problems in space mostly include the application of topical creams. Moisturizing cream was used on one arm of an astronaut measuring skin parameters in space. The treatment with this skin care emulsion improved the hydration and barrier function of the epidermis compared with the untreated side [16]. The skin problems observed after return from a one-year mission in another astronaut were successfully treated on Earth, using nonsteroidal anti-inflammatory drugs, gabapentin, hydrotherapy, and massage [12]. Attempts in resolving the delayed wound healing observed in microgravity has shown promising results in a leech (*Hirudo medicinalis)* model of wound healing, where delayed wound healing induced by simulated microgravity could be successfully counteracted by treatment with platelet-rich plasma [98]. In the case of slow-healing open wounds, wound dressings can support healing of the wound and protect it from infections. Hydrogels, which are gel-like materials capable of holding large volumes of water, provide suitable candidates for wound dressings, as the moist environment can promote healing [99]. A novel technique that holds promising expectations for future healthcare in space is tissue nanotransfection technology, which reprograms in vivo tissues and cells (e.g., fibroblasts in skin) to induce neurons and endothelial cells [100]. Furthermore, healthcare in space can benefit from drug delivery through transdermal polymeric systems, as this passive system requires no invasive medical procedures, and is easily self-administered [101].

In conclusion, long-term exposure to the spaceflight environment is associated with skin health problems, increasing the risk of losing vital skin functions during future long-term space exploration. Simulation models on Earth are needed to investigate the effects of individual spaceflight stressors of cosmic radiation, microgravity and psychological stress hormones. Moreover, the combination of such simulation models are needed to identify possible interaction effects, to improve the risk assessment associated with future deep-space travel, and give insights into the development of effective countermeasures.

## Figures and Tables

**Table 2:** Summary of ion exposure conditions of discussed studies *.

Ion	Energy [MeV/u]	LET [keV/µm]	Findings	Reference
Carbon	135	38; 55; 84; 91; 94; 98	Ion species-dependent differences in energy deposition track structures affect cell killing	[58]
	290	13; 19; 38; 54; 64; 73; 76; 80		
Neon	230	45; 59; 77; 105; 132; 158; 177		
	400	30; 44; 58; 77; 105; 127; 156; 184		
Silicon	490	55; 59; 69; 113; 145; 173; 214		
Iron	500	200; 260; 300; 350; 400		
Oxygen	55	77	Non-targeted effect for chromosomal aberrations in fibroblasts exposed to low-particle fluences	[61]
Silicon	170	99		
Iron	300	240		
	450	195		
	600	175		
Neon	400	54	Changes in the gene expression of fibroblasts increased with particle energy	[63]
Silicon	490	55		
Iron	1000	145		
Iron	1010	-	Exposure of rat keratinocytes to 3 Gy of iron ions increased gene expression profile related to cell cycle which were linked to reproductive cell death	[64]
Carbon	-	110–145	Pro-inflammatory signals, homeostasis and epidermal tissue organization changes were observed after carbon ion exposure	[66]
Oxygen	500	18	3D organotypic in vitro skin model proliferation and differentiation profiles are affected by low-dose ion irradiation	[67]
Neon	300	35		
Silicon	400	60		
Iron	600	174		

* Gy, Gray; LET, linear energy transfer; MeV/u, megaelectron volts per nucleon.
